# Supplementary material for: MetaRibo-Seq measures translation in microbiomes
Source: Nat Commun. 2020 Jun 29;11:3268. doi: 10.1038/s41467-020-17081-z (PMC7324362; doi:10.1038/s41467-020-17081-z)
Supplement: Supplementary file 10 — Supplementary Data 7 [file 41467_2020_17081_MOESM10_ESM.zip › File2/Confidence_High_Taxonomy/306475_out.krona.html]

Javascript must be enabled to view this page.

members
magnitude
magnitudeUnassigned
count
unassigned
taxon
rank

306475\_out

34

2
superkingdom
34

34
976
phylum

34
class
200643

34
171549
order

family
815
2

2
genus
816


SRS050299\_contig\_number\_2754
species
246787
1

1
species
47678

SRS019030\_contig\_number\_10921

32
family
2005525

375288
genus
32


SRS013098\_contig\_number\_38875SRS015264\_contig\_number\_12183SRS016018\_contig\_number\_12270SRS016132\_contig\_number\_4670SRS016753\_contig\_number\_1819SRS019445\_contig\_number\_28624SRS022524\_contig\_number\_14033SRS023583\_contig\_number\_contig-100\_841.106456SRS042628\_contig\_number\_38634SRS043667\_contig\_number\_5167SRS045739\_contig\_number\_2160SRS047433\_contig\_number\_21948SRS063040\_contig\_number\_15092SRS065397\_contig\_number\_contig-100\_352.82079SRS074670\_contig\_number\_3643SRS075821\_contig\_number\_21118SRS077086\_contig\_number\_5119SRS078419\_contig\_number\_26627SRS097889\_contig\_number\_34377SRS098571\_contig\_number\_56974SRS104036\_contig\_number\_8027SRS104975\_contig\_number\_21017SRS1055022\_contig\_number\_22381SRS140513\_contig\_number\_3612SRS142923\_contig\_number\_contig-100\_317.112689SRS143780\_contig\_number\_19562SRS144506\_contig\_number\_45007SRS146764\_contig\_number\_15470SRS146813\_contig\_number\_15016SRS147088\_contig\_number\_contig-100\_6119.6119SRS147346\_contig\_number\_64865SRS148424\_contig\_number\_16840
46503
species
32
